# Rubi Fructus Water Extract Alleviates LPS-Stimulated Macrophage Activation via an ER Stress-Induced Calcium/CHOP Signaling Pathway

**DOI:** 10.3390/nu12113577

**Published:** 2020-11-22

**Authors:** Do-Hoon Kim, Ji-Young Lee, Young-Jin Kim, Hyun-Ju Kim, Wansu Park

**Affiliations:** 1 Department of Medical Classics and History, College of Korean Medicine, Gachon University, Seongnam 13120, Korea; chulian@gachon.ac.kr; 2 Department of Pathology, College of Korean Medicine, Gachon University, Seongnam 13120, Korea; oxygen1119@naver.com (J.-Y.L.); godsentry@naver.com (Y.-J.K.); eternity0304@daum.net (H.-J.K.)

**Keywords:** Rubi Fructus, Rubus coreanus, lipopolysaccharide, macrophage, ER stress, calcium, chop, STAT, cytokine, nitric oxide

## Abstract

Despite the availability of antibiotics and vaccines, many intractable infectious diseases still threaten human health across the globe. Uncontrolled infections can lead to systemic inflammatory response syndrome and the excessive production of inflammatory cytokines, known as a cytokine storm. As cytokines also play necessary and positive roles in fighting infections, it is important to identify nontoxic and anti-inflammatory natural products that can modulate cytokine production caused by infections. Rubi Fructus, the unripe fruits of *Rubus coreanus* Miquel, are known to possess antioxidative properties. In this study, the effect of the water extract of Rubi Fructus (RF) on the lipopolysaccharide (LPS)-induced inflammatory response in RAW 264.7 macrophages was investigated using biochemical and cell biology techniques. Our data indicated that RF inhibits p38 phosphorylation, intracellular calcium release, and the production of nitric oxide (NO), interleukin (IL)-6, monocyte chemotactic activating factor (MCP)-1, tumor necrosis factor (TNF)-α, leukemia inhibitory factor (LIF), lipopolysaccharide-induced CXC chemokine (LIX), granulocyte-colony stimulating factor (G-CSF), granulocyte macrophage colony-stimulating factor (GM-CSF), vascular endothelial growth factor (VEGF), macrophage colony-stimulating factor (M-CSF), macrophage inflammatory protein (MIP)-1α, MIP-1β, MIP-2, and regulated on activation, normal T cell expressed and secreted (RANTES) in LPS-treated macrophages. In addition, we observed decreasing mRNA expression of *Chop*, *Camk2a*, *Stat1*, *Stat3*, *Jak2*, *Fas*, *c-Jun*, *c-Fos*, *Nos2*, and *Ptgs2* without cytotoxic effects. We concluded that RF demonstrated immunoregulatory activity on LPS-stimulated macrophages via an endoplasmic reticulum (ER) stress-induced calcium/CCAAT-enhancer-binding protein homologous protein (CHOP) pathway and the Janus kinase (JAK)/signal transducers and activators of transcription (STAT) pathway.

## 1. Introduction

Inflammatory reactions in response to pathogenic infections are essential for human survival [1]. These inflammatory cascades are regulated by immune cells, such as neutrophils, monocytes, macrophages, dendritic cells, eosinophils, basophils, T-lymphocytes, and B-lymphocytes [2]. Among these cell types, macrophages are one of the major regulators of the innate immune system response to infectious pathogens [3]. Macrophages are well known to identify and destroy intrusive microorganisms (i.e., gram-negative bacteria) via the upregulation of inflammatory mediators, such as nitric oxide (NO), cytokines, chemokines, growth factors, prostaglandins, leukotrienes, and blood coagulation factors [4]. One of major pathways induced by endotoxins, such as lipopolysaccharide (LPS), is the endoplasmic reticulum (ER) stress-induced calcium/CCAAT-enhancer-binding protein homologous protein (CHOP) pathway, which consists of calcium release from the NO-stressed ER and activation of CHOP, calcium/calmodulin dependent protein kinase II alpha (CAMK2a), signal transducers and activators of transcription (STAT), and Fas proteins [5]. However, although necessary for the removal of invasive pathogens, macrophage activation can lead to a cytokine storm (hypercytokinemia), or the excessive production of cytokines, commonly observed in systemic inflammatory response syndrome (SIRS), resulting in multiple organ dysfunction [6]. As there are no effective therapies for cytokine storm, it is important to search for nontoxic and anti-inflammatory natural products that can modulate cytokine production caused by infections.

Traditional medicines can be beneficial for human health, as reported by Tu Youyou, who was awarded the 2015 Nobel Prize for Physiology or Medicine for the discovery of artemisinin from *Artemisia apiacea* [7]. Rubi Fructus (Black Raspberry), the unripe fruits of *Rubus coreanus* Miquel, has traditionally been used as a medical drug in East Asia, including Korea and China [8,9]. Rubi Fructus is also well known to have antioxidative properties [10] and contain large quantities of polyphenolic compounds [11]. Concretely, Seo et al. reported in 2019 that the ethanol extract of Rubi Fructus (ERF) demonstrated that high radical scavenging activity and inhibited the production of inflammatory mediators via the nuclear factor (NF)-κB signaling pathway in RAW 264.7 macrophages stimulated by lipopolysaccharide (LPS) [12]. In 2014, Lee et al. reported that ERF reduced the production of inflammatory mediators, such as NO, prostaglandin E2 (PGE2), tumor necrosis factor (TNF)-α, interleukin (IL)-1β, and IL-6 via suppression of NF-κB and mitogen-activated protein kinase (MAPK) activation in LPS-stimulated RAW 264.7 cells [13]. In 2013, Kim et al. reported that the water extract of Rubi Fructus (WRF) also suppressed NF-κB activation, reactive oxygen species (ROS) production, and inflammatory and phase II gene expression in LPS-stimulated RAW 264.7 macrophages [14].

In the previous study, we reported that *Angelica sinensis* root water extract has an anti-inflammatory effect on LPS-stimulated RAW 264.7 via calcium-mediated Janus kinase (JAK)-STAT pathway [15]. Since Rubi Fructus has antioxidative properties, such as *Angelica sinensis* root, we set the hypothesis that Rubi Fructus modulates inflammatory reactions in LPS-stimulated macrophages via calcium-STAT signaling pathway and conducted related experiments to evaluate effects of the WRF (RF) on the inflammatory cascade in LPS-stimulated RAW 264.7. Experimental data showed that RF inhibited p38 MAPK phosphorylation, intracellular calcium release, and production of NO and various cytokines, chemokines, and growth factors in LPS-stimulated RAW 264.7 cells. We also detected decreased mRNA expressions of *Chop, Camk2a, Stat1, Stat3, Jak2, Fas, c-Jun, c-Fos, Nos2,* and *Ptgs2* without cytotoxic effect. These results indicate that RF possesses immunoregulatory activity in LPS-stimulated macrophages via ER stress-induced calcium/CHOP pathway and the JAK/STAT pathway.

## 2. Materials and Methods

### 2.1. Materials

Dulbecco’s modified Eagle medium (DMEM), LPS (0.1~1 µg/mL), baicalein (25 µM), and other cell culture reagents were obtained from Millipore (Billerica, MA, USA). Phospho-p38 MAPK Antibody (T180/Y182) (eBioscience 17-9078-42) and Mouse immunoglobulin G2b (IgG2b) kappa Isotype Control (eBioscience 12-4732-81) were obtained from Life Technologies Corporation (Carlsbad, CA, USA).

### 2.2. Preparation of RF

Commercial Rubi Fructus were obtained from Omniherb (Daegu, Korea) and authenticated by Professor W. Park of Gachon University in July 2016. A voucher specimen (no. 2016-0012) was deposited at the Department of Pathology in Gachon University’s College of Korean Medicine. As herbal drugs have been traditionally extracted using water, in the present study, Rubi Fructus were extracted with boiling water for 2 h, filtered, and then lyophilized (yield: 17.42%). The powdered extract (25~200 mg/mL) was dissolved in saline and then filtered through a 0.22 µm syringe filter [15].

### 2.3. Total Flavonoid Content of RF

The total flavonoid content of RF was determined using the diethylene glycol colorimetric method. Briefly, the sample solution (20 µL of 2 mg/mL RF) was mixed with 200 µL of diethylene glycol and 20 µL of 1 N NaOH. The sample absorbance was read at 405 nm after 1 h incubation at 37 °C. Rutin was used as a reference standard, and total flavonoid content was expressed as milligrams of rutin equivalents (mg RE/g extract) [16].

### 2.4. Effects of RF on Cell Viability of RAW 264.7

RAW 264.7 mouse macrophages were purchased from the Korea Cell Line Bank (Seoul, Korea). RAW 264.7 cells were cultured in DMEM supplemented with 10% fetal bovine serum containing 100 U/mL of penicillin and 100 µg/mL of streptomycin at 37 °C in a 5% CO_2_ humidified incubator. Cell viability was evaluated using a modified MTT assay in 96-well plates (1 × 10^4^ cells/well). Optical density (OD) was determined at 540 nm with a microplate reader (Bio-Rad, Hercules, CA, USA) [15]. In order to determine the toxicity of RF, RF at concentrations of 25, 50, 100, or 200 µg/mL were used for the dose response experiments.

### 2.5. Effects of RF on NO Production in RAW 264.7 Macrophages Stimulated with LPS

The concentration of NO in culture medium was determined using a Griess reaction assay. Specifically, after incubation of cells in 96-well plates (1 × 10^4^ cells/well) for 24 h with LPS (1 µg/mL) and RF, 100 µL of supernatant from each well was collected and mixed with 100 µL of Griess reagent in a new 96-well plate. After an incubation of 15 min at room temperature, OD was determined at 540 nm with a microplate reader (Bio-Rad) [15].

### 2.6. Effects of RF on Intracellular Calcium Release in RAW 264.7 Stimulated by LPS

After RAW 264.7 cells were seeded in 96-well plates (1 × 10^5^ cells/well), LPS (1 µg/mL) and RF were added to the culture medium and incubated for 18 h at 37 °C. Thereafter, the medium was removed and cells were incubated with 100 µL of the Fluo-4 dye loading solution (Molecular Probes, Eugene, OR, USA) for 30 min at 37 °C. After 30 min incubation, cells were incubated for a further 30 min at room temperature. Then, the fluorescence intensity of each well was determined using a spectrofluorometer (Dynex, West Sussex, UK) at excitation and emission wavelengths of 485 nm and 535 nm, respectively [15].

### 2.7. Effects of RF on Cytokine Production in RAW 264.7 Cells Stimulated by LPS

RAW 264.7 cells were seeded in 96-well plates (1 × 10^4^ cells/well) and treated with LPS (1 µg/mL) and RF [15]. After 24 h treatment, levels of the following cytokines in each well were analyzed: interleukin (IL)-6; monocyte chemoattractant protein (MCP)-1; tumor necrosis factor (TNF)-α; leukemia inhibitory factor (LIF); lipopolysaccharide-induced CXC chemokine (LIX; CXCL5); granulocyte colony-stimulating factor (G-CSF); granulocyte macrophage colony-stimulating factor (GM-CSF); macrophage colony-stimulating factor (M-CSF); vascular endothelial growth factor (VEGF); macrophage inflammatory proteins (MIP)-1α, MIP-1β, MIP-2; RANTES (CCL5; regulated on activation, normal T cell expressed and secreted); and interferon gamma-induced protein 10 (IP-10; CXCL10). Cytokines were measured using a Luminex assay based on xMAP technology with MILLIPLEX MAP Mouse Cytokine/Chemokine Magnetic Bead Panel kits (Millipore) and a Bio-Plex 200 suspension array system (Bio-Rad), as described previously [15]. The assay used in this experiment was designed for the multiplexed quantitative measurement of multiple cytokines in a single well, using as little as 25 µL of cell culture supernatant. Standard curves for each cytokine were generated using the kit-supplied reference cytokine samples. Briefly, the following procedure was performed: after pre-wetting the 96-well plate with Wash Buffer, Wash Buffer in each well was removed using a Handheld Magnetic Separation Block (HMSB). Next, cell culture supernatants from each well were incubated with antibody-conjugated beads on a plate shaker for 2 h at room temperature. After incubation, well contents were gently removed with a HMSB, and the 96-well plate was washed 2 times. Then, 25 µL of detection antibodies were added to each well and incubated with agitation on a plate shaker for 1 h at room temperature. Subsequently, 25 µL Streptavidin–Phycoerythrin was added to each well containing the detection antibodies and incubated for 30 min with agitation on a plate shaker at room temperature. After incubation, the well contents were gently removed and washed 2 times using a HMSB. Then, 150 µL of Sheath Fluid was added to all wells, and the beads bound to each cytokine were analyzed with a Bio-Plex 200 instrument (Bio-Rad). Raw data (fluorescence intensity) were analyzed using Bio-Plex Manager software (Bio-Rad). Baicalein (25 µM), a well-known anti-inflammatory flavonoid, was used as a positive control.

### 2.8. Effects of RF on mRNA Expression in RAW 264.7 Cells Stimulated by LPS

#### 2.8.1. Isolation of RNA

RAW 264.7 cells were incubated with LPS (1 µg/mL) and RF for 18 h in 6-well plates (1 × 10^6^ cells/well). After 18 h incubation, total RNA of each well was isolated using NucleoSpin RNA kit (Macherey-Nagel, Duren, Germany). Briefly, 350 μL Lysis Buffer RA1 and 3.5 μL β-mercaptoethanol was added to the cell pellet and vortexed vigorously to lyse cells. Lysate was cleared by filtration using a NucleoSpin^®^ Filter, and then 350 μL ethanol (70%) was added, and mixed by vortexing. The lysate was loaded into the NucleoSpin^®^ RNA Column, and 350 μL Membrane Desalting Buffer was added and centrifuged. A total of 95 μL DNase reaction mixture was applied directly to the center of the silica membrane of the column, followed by incubation at room temperature for 15 min. Samples were washed with Wash Buffer RA2 and Wash Buffer RA3, and silica membrane was dried. RNA was eluted in 60 μL RNase-free water and centrifuged [15].

#### 2.8.2. Determination of RNA Concentration

RNA concentration was measured using Experion RNA StdSens Analysis kit (Bio-Rad) with the Experion Automatic Electrophoresis System (Bio-Rad). First, the electrodes were cleaned using a cleaning chip filled with 900 μL DEPC-treated water. Then, the Gel-Stain solution was prepared, 9 μL was added into labeled wells, and the chip was primed. Samples and RNA ladder were loaded into the chip, which was vortexed using the Experion vortex station for 1 min. Then the chip was loaded into the electrophoresis platform and the RNA StdSens Analysis program was run [15].

#### 2.8.3. cDNA Synthesis

cDNA of the RNA samples was produced using iScript cDNA Synthesis kit (Bio-Rad) [15]. Briefly, 20 μL complete reaction mixes were prepared with 5× iScript Reaction Mix (4 μL), iScript Reverse Transcriptase (1 μL), Nuclease-free water (variable), and RNA template (variable, 1 μg total RNA). The reaction mix (20 μL) was incubated in a thermal cycler (C1000 Thermal Cycler, Bio-Rad) according to the manufacturer’s protocol (priming at 25 °C for 5 min, reverse transcription at 46 °C for 20 min, and RT inactivation at 95 °C for 1 min).

#### 2.8.4. RT-qPCR Analysis

Gene expression was measured using quantitative polymerase chain reaction with iQ SYBR Green Supermix (Bio-Rad) using the CFX96 Real-Time PCR Detection System (Bio-Rad) [15]. Briefly, a master mix was prepared for all reactions by adding iQ SYBR Green Supermix and Forward/Reverse primers for each target gene. This master mix was thoroughly mixed to ensure homogeneity, and 7 μL was dispensed into the wells of a qPCR plate. A total of 3 μL of cDNA was added to each well; any air bubbles in the vessel bottom were removed, and the PCR plate was loaded into the real-time PCR instrument. PCR was performed using the following protocol: denaturation of DNA at 95 °C for 3 min, followed by 40 cycles of 95 °C for 10 sec and 55 °C for 30 sec. The 2^–Δ∆Ct^ cycle threshold method was used to normalize the relative mRNA expression levels to the internal control, *β-actin*. The primers used in this assay are listed in Table 1.

### 2.9. Effects of RF on Phosphorylation of p38 MAPK in RAW 264.7 Cells Stimulated by LPS

Flow cytometry was performed to detect phosphorylated p38 MAPK in RAW 264.7 cells using an Attune NxT flow cytometer (Thermo Fisher Scientific) [17]. Briefly, RAW 264.7 macrophages were seeded in 6-well plates (1 × 10^6^ cells/well) and incubated with LPS (0.1 µg/mL) and RF for 30 min. After incubation, cells were harvested and washed with Flow Cytometry Staining Buffer (SB). Prior to antibody staining, cells were fixed with the pre-warmed Fix Buffer I for 10 min. Then, cells were washed with SB and permeabilized with Perm Buffer III on the ice for 30 min. Then, cells were stained with 5 µg/mL of phospho-p38 MAPK Antibody (T180/Y182) (eBioscience 17-9078-42), or 1.2 µg/mL of Mouse IgG2b kappa Isotype Control (eBioscience 12-4732-81), and analyzed on the Attune NxT flow cytometer (Thermo Fisher Scientific) using Attune NxT software (Thermo Fisher Scientific).

### 2.10. Statistics

Data are presented as means ± SD. All data were analyzed by one-way analysis of variance (ANOVA) test followed by Tukey’s multiple comparison test using GraphPad Prism (version 4; GraphPad Software, San Diego, CA, USA).

## 3. Results

### 3.1. Determination of the Total Flavonoid Content of RF

We found that the total flavonoid content of RF was 9.15 mg RE/g extract.

### 3.2. Effects of RF on Cell Viability

**Figure 1:**
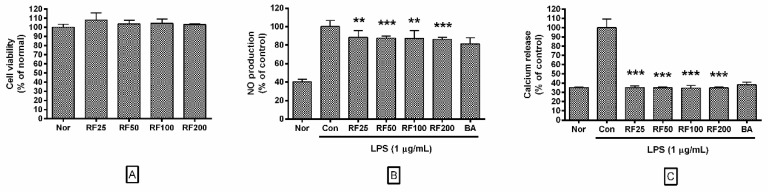
Effects of Rubi Fructus water extract (RF) on (**A**) cell viability, (**B**) nitric oxide (NO) production, and (**C**) intracellular calcium release. Cells were treated with RF and LPS for 24 h (**A**,**B**) or 18 h (**C**). “Nor” indicates the group treated with media only. “Con” indicates the group treated with 1 µg/mL of lipopolysaccharide (LPS) alone. RF25, RF50, RF100, and RF200 indicate 25, 50, 100, and 200 µg/mL of RF, respectively. “BA” indicates treatment with baicalein (25 µM). Values represent means ± SD of three independent experiments (*n* = 3). Statistical significance was calculated by one-way ANOVA and a Tukey multiple comparison test. ** *p* < 0.01 vs. Con; *** *p* < 0.001 vs. Con.

In this study, RF at concentrations of 25, 50, 100, or 200 µg/mL did not decrease cell viability of RAW 264.7 cells after 24 h (108.00 ± 7.84%, 103.84 ± 3.71%, 104.47 ± 4.77%, and 103.19 ± 0.83% of the normal group (Nor) treated with media only, respectively). These results indicated that RF does not exert any cytotoxic effect on macrophages at concentrations of up to 200 µg/mL, which were used in all subsequent experiments (Figure 1A).

### 3.3. NO Production in RAW 264.7 Cells

Data showed that RF significantly inhibited NO production from RAW 264.7 cells stimulated by 24 h treatment with LPS. NO production in RAW 264.7 cells incubated with RF at concentrations of 25, 50, 100, and 200 µg/mL were 88.29 ± 7.33%, 87.6 ± 2.22%, 87.22 ± 8.5%, and 86.26 ± 1.98% of that treated with LPS alone (Figure 1B). These data indicated that RF might modulate excessive NO-induced inflammatory signaling.

### 3.4. Calcium Release in RAW 264.7 Cells

Data showed that RF significantly inhibited calcium release in RAW 264.7 cells stimulated by 18 h LPS treatment. Calcium release in RAW 264.7 cells incubated with RF at concentrations of 25, 50, 100, and 200 µg/mL were 35.25 ± 1.86%, 34.91 ± 1.12%, 34.72 ± 2.6%, and 34.94 ± 1.08% of that induced by LPS treatment alone (Figure 1C). Our results indicated that RF might exert a regulatory effect over the calcium-related ER stress response pathway.

### 3.5. Cytokine Production in RAW 264.7 Cells

**Figure 2:**
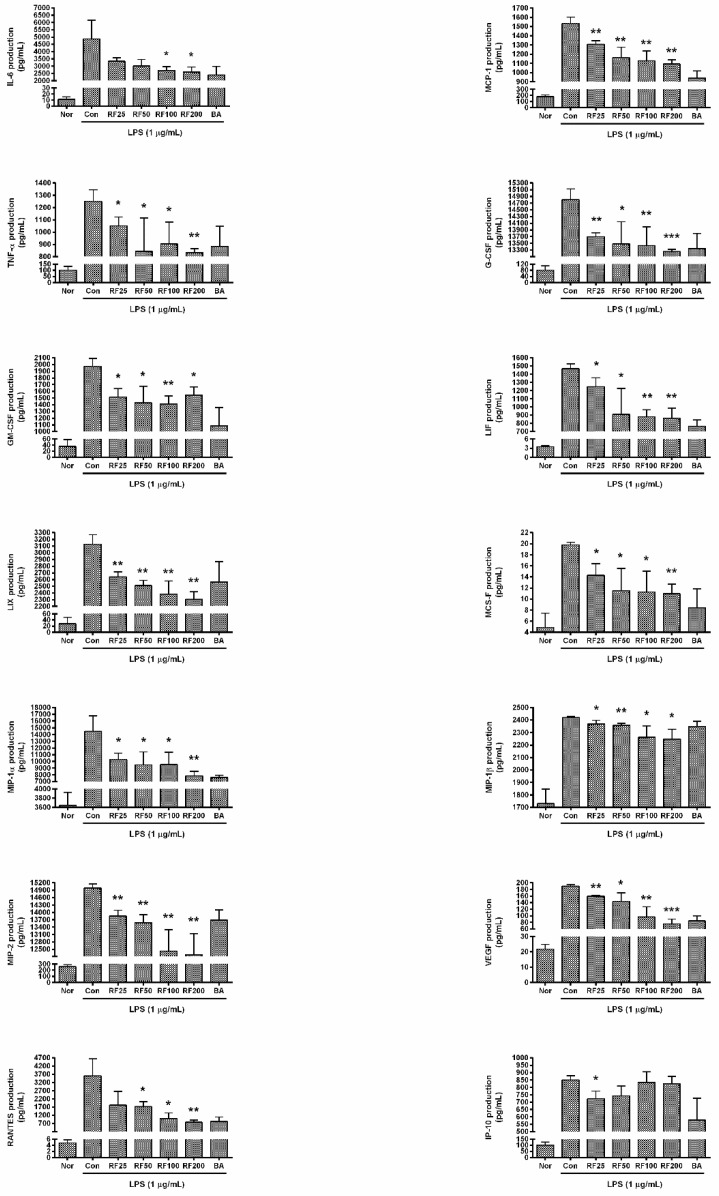
Effects of Rubi Fructus water extract (RF) on the production of interleukin (IL)-6, monocyte chemotactic activating factor (MCP)-1, tumor necrosis factor (TNF)-α, granulocyte-colony stimulating factor (G-CSF), granulocyte macrophage colony-stimulating factor (GM-CSF), leukemia inhibitory factor (LIF), lipopolysaccharide-induced CXC chemokine (LIX), macrophage colony-stimulating factor (M-CSF), macrophage inflammatory protein (MIP)-1α, MIP-1β, MIP-2, vascular endothelial growth factor (VEGF), and regulated on activation, normal T cell expressed and secreted (RANTES), and interferon gamma-induced protein (IP)-10. Cells were treated for 24 h with LPS and RF. “Nor” indicates the group treated with media only. “Con” indicates the group treated with 1 µg/mL of LPS alone. RF25, RF50, RF100, and RF200 indicate 25, 50, 100, and 200 µg/mL of RF, respectively. “BA” indicates treatment with baicalein (25 µM). Values represent means ± SD of three independent experiments (*n* = 3). Statistical significance was calculated by one-way ANOVA and a Tukey multiple comparison test. * *p* < 0.05 vs. Con; ** *p* < 0.01 vs. Con; *** *p* < 0.001 vs. Con.

RF significantly reduced the production of cytokines in RAW 264.7 cells stimulated by LPS for 24 h. In particular, RF reduced productions of IL-6, MCP-1, G-CSF, LIF, LIX, MIP-1α, MIP-1β, MIP-2, VEGF, and RANTES in a dose-dependent manner. Production of IL-6 from RAW 264.7 cells with RF (25, 50, 100, and 200 µg/mL) were 90.71 ± 2.86%, 86.09 ± 6.48%, 81.58 ± 4.52%, and 79.675%± 5.9% of the LPS alone, respectively; production of MCP-1 was 77.65 ± 3.81%, 64.67 ± 10.02%, 61.73 ± 8.88%, and 58.47 ± 4.14%, respectively; TNF-α was 87.31 ± 3.81%, 69.78 ± 22.36%, 80.5 ± 14.54%, and 76.73 ± 12.05%, respectively; G-CSF was 95.79 ± 0.45%, 94.92 ± 2.63%, 94.73 ± 2.19%, and 94.03 ± 0.24%, respectively; GM-CSF was 57.76 ± 4.91%, 52.2 ± 8.32%, 57.44 ± 7.98%, and 66.48 ± 8.63%, respectively; LIF was 89.1 ± 5.63%, 72.43 ± 17%, 68.2 ± 5.4%, and 67.06 ± 7.78%, respectively; LIX was 80.23 ± 2.96%, 75.11 ± 3.12%, 70.3 ± 7.42%, and 67.26 ± 4.23%, respectively; M-CSF was 84.55 ± 5.85%, 72.87 ± 8.2%, 76.49 ± 9.85%, and 75.45 ± 4.67%, respectively; MIP-1α was 94.8 ± 1.14%, 93.8 ± 0.75%, 93.12 ± 1.57%, and 92.02 ± 1.21%, respectively; MIP-1β was 97.85 ± 1.2%, 97.42 ± 1.2%, 93.52 ± 3.66%, and 92.93 ± 3.22%, respectively; MIP-2 was 97.37 ± 0.57%, 96.69 ± 0.81%, 93.58 ± 2.42%, and 93.16 ± 2.45%, respectively; VEGF was 85.71 ± 1.25%, 77.54 ± 12.82%, 53.28 ± 16.42%, and 41.97 ± 7.72%, respectively; RANTES was 92.07 ± 13.19%, 89.13 ± 3.04%, 77.7 ± 7.13%, and 71.77 ± 5.62%, respectively; and IP-10 was 84.42 ± 5.19%, 90.04 ± 6.85%, 99.26 ± 7.16%, and 98.33 ± 5%, respectively (Figure 2). These data indicated that RF might alleviate hyper-inflammation leading to excessive production of cytokines, chemokines, and growth factors in LPS-stimulated macrophages.

### 3.6. mRNA Expression in RAW 264.7 Cells

**Figure 3:**
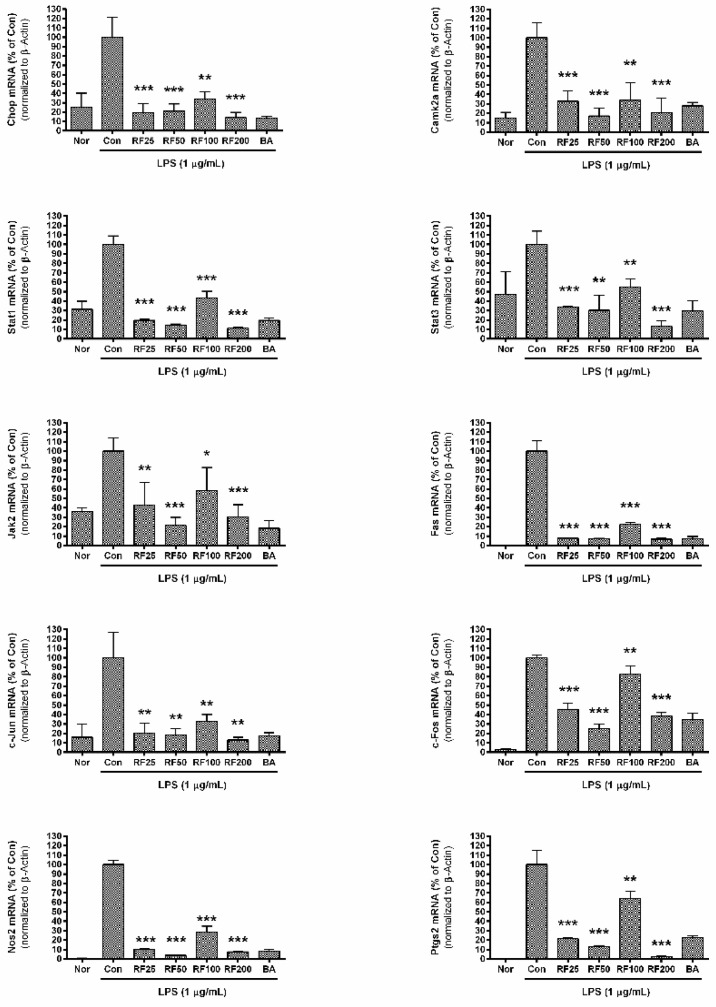
Effects of Rubi Fructus water extract (RF) on mRNA expression of *Chop, Camk2a, Stat1, Stat3, Jak2, Fas, c-Jun, c-Fos, Nos2,* and *Ptgs2*. Cells were treated for 18 h. “Nor” indicates the group treated with media only. “Con” indicates the group treated with 1 µg/mL of LPS alone. RF25, RF50, RF100, and RF200 indicate treatment with 25, 50, 100, and 200 µg/mL of RF, respectively. “BA” indicates treatment with baicalein (25 µM). Values represent means ± SD of three independent experiments (*n* = 3). Statistical significance was calculated by one-way ANOVA and a Tukey multiple comparison test. * *p* < 0.05 vs. Con; ** *p* < 0.01 vs. Con; *** *p* < 0.001 vs. Con.

RF significantly inhibited the mRNA expression of *Chop, Camk2a, Stat1, Stat3, Jak2, Fas, c-Jun, c-Fos, Nos2,* and *Ptgs2* in RAW 264.7 cells stimulated by LPS (Figure 3). *Chop* expression in RAW 264.7 cells incubated with RF (25, 50, 100, and 200 µg/mL) were 19.35 ± 9.77%, 21.06 ± 7.78%, 33.91 ± 7.73%, and 14.25 ± 5.6% of LPS-treated cells alone, respectively; *Camk2a* expression was 32.76 ± 10.69%, 17.08 ± 8.21%, 33.85 ± 18.44%, and 20.78 ± 15.3% of the LPS group alone; *Stat1* was 19.52 ± 1.36%, 14.73 ± 0.94%, 43.41 ± 7.07%, and 11.36 ± 1.32%, respectively; *Stat3* was 33.76 ± 1.1%, 30.23 ± 16.19%, 54.86 ± 8.74%, and 13.22 ± 5.66%, respectively; *Jak2* was 42.92 ± 23.87%, 21.27 ± 8.54%, 58.1 ± 24.55%, and 30.63 ± 12.68%, respectively; *Fas* was 7.71 ± 0.71%, 7.01 ± 0.55%, 22.49 ± 2.15%, and 6.79 ± 1.15%, respectively; *c-Jun* was 20.29 ± 10.56%, 18.4 ± 6.7%, 32.78 ± 7.4%, and 13.08 ± 2.87%, respectively; *c-Fos* was 45.53 ± 6.31%, 24.94 ± 5.15%, 82.64 ± 8.96%, and 38.25 ± 3.68%, respectively; *Nos2* was 10.44 ± 0.91%, 3.89 ± 0.36%, 28.42 ± 6.43%, and 7.63 ± 0.55%, respectively; and *Ptgs2* was 21.47 ± 0.66%, 13.04 ± 1.17%, 64.17 ± 7.6%, and 2.66 ± 0.82%, respectively. Although RF did not exert a concentration-dependent inhibition of mRNA expression, these data indicate that RF might modulate the expression of inflammatory genes related to ER stress.

### 3.7. Phosphorylation of p38 MAPK in RAW 264.7

**Figure 4:**
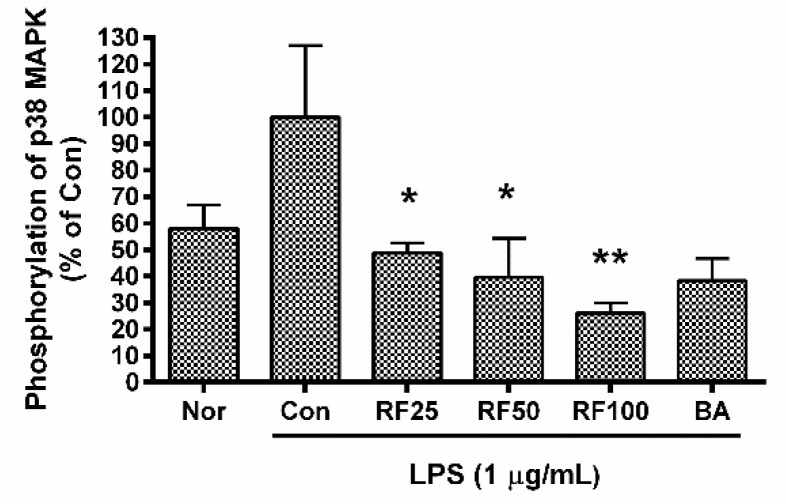
Effects of Rubi Fructus water extract (RF) on phosphorylation of p38 MAPK in RAW 264.7 cells. Cells were treated with LPS and RF for 30 min. “Nor” indicates the group treated with media only. “Con” indicates the group treated with 0.1 µg/mL of LPS alone. RF25, RF50, RF100, and RF200 indicate treatment with 25, 50, 100, and 200 µg/mL of RF, respectively. “BA” indicates treatment with baicalein (25 µM). Values represent means ± SD of three independent experiments (*n* = 3). Statistical significance was calculated by one-way ANOVA and a Tukey multiple comparison test. * *p* < 0.05 vs. Con; ** *p* < 0.01 vs. Con.

Phosphorylation of p38 MAPK was significantly inhibited by RF (Figure 4). p38 MAPK phosphorylation in RAW 264.7 cells incubated with RF (25, 50, 100, and 200 µg/mL) for 30 min was 48.76 ± 3.79%, 39.64 ± 14.53%, 26.11 ± 3.87%, and 38.29 ± 8.46% of that induced by LPS. No non-specific staining was observed with Mouse IgG2b kappa Isotype Control. These data indicated that RF might exert its anti-inflammatory effects via suppressing the activation of p38 MAPK signaling pathway.

## 4. Discussion

SIRS caused by gram-negative bacteria is known to be a host response to lipopolysaccharide [18,19] and accompanied by increased production of inflammatory mediators such as NO, IL-1, IL-6, and TNF-α resulting in vascular leakage and multiple organ dysfunction syndrome (MODS) [20,21]. Increased endothelial permeability is central to SIRS, leading to MODS and death [22,23,24,25]. Indeed, in addition to the cytopathic role in host defense and innate immune functions, NO plays a major role in vasodilation in SIRS [26]. Despite a definitive link between cytokine levels and morbidity/mortality following infection, no effective therapeutic modalities have been developed to subdue the pathology associated with cytokine storm [27]. However, because of the necessary and positive function of cytokines against pathogenic infections, global blunting, rather than ablation, of inflammatory mediators will likely be required to ameliorate pathology associated with cytokine storm [27]. Among immune cells, macrophages are well known to produce many kinds of inflammatory mediators, such as NO and cytokines. Therefore, a drug candidate able to modulate the excessive activation of macrophages stimulated by LPS, including the excessive production of NO and cytokines, might be a beneficial for the regulation of endotoxemia-related inflammatory cascades.

Until recently, many studies have reported on the anti-inflammatory effects of natural products. In the previous study, we reported that *Angelica sinensis* root water extract has an anti-inflammatory effect on LPS-stimulated RAW 264.7 via calcium-mediated JAK-STAT pathway [15]. Since Rubi Fructus has also antioxidative properties like *Angelica sinensis* root, we set the hypothesis that Rubi Fructus inhibits inflammatory reactions in LPS-stimulated macrophages via calcium-STAT signaling pathway. Actually, Rubi Fructus has traditionally been used as a medical drug in East Asia, including Korea and China [8,9]. While the use of Rubi Fructus is indicated in textbooks of traditional medicine such as “Donguibogam (Principles and Practice of Eastern Medicine)” and “Ben Cao Gang Mu (Compendium of Materia Medica)”, many researchers are interested in investigating the pharmacological activities of Rubi Fructus [8,9]. A number of studies have reported on the biomedical efficacy of Rubi Fructus, such as its anti-inflammatory effects [12], antioxidative effects [10], improvements in visual sensitivity [28], acetylcholinesterase inhibitory activity [29], improvements in diabetic osteoporosis by simultaneous regulation of osteoblasts and osteoclasts [30], hepatoprotective effects [31], anti-fatigue effects [32], increased hypocholesterolemic activity [33], chemopreventive effects in prostate cancer [34], increased anti-anaphylactic activity [35], and enhanced spermatogenesis [36]. In detail, Seo et al. reported in 2019 that ERF inhibited the production of NO, IL-1β, and IL-6 as well as the activation of inducible nitric oxide synthase (iNOS) and cyclooxygenase 2 (COX-2) via inhibition of the NF-κB signaling pathway in LPS-stimulated RAW 264.7 [12]. Lee et al. reported in 2014 that ERF reduced production of NO, PGE2, TNF-α, IL-1β, and IL-6, and reduced expression of iNOS and COX-2 through suppression of NF-κB activation, as well as phosphorylation of JNK and p38 MAPKs [13]. Kim et al. reported in 2013 that the WRF suppressed NF-κB activation, ROS production, and inflammatory and phase II gene expression in LPS-treated RAW 264.7 cells [14]. Additionally, Park et al. reported in 2006 that ERF exerts anti-inflammatory effects in macrophages via activation of the heme oxygenase-1 signaling pathway [37].

In these experiments, RF significantly inhibited excessive production of NO, IL-6, TNF-α, MCP-1, LIF, LIX, RANTES, MIP-1α, MIP-1β, MIP-2, G-CSF, GM-CSF, VEGF, and M-CSF in RAW 264.7 macrophages stimulated by LPS. The half maximal inhibitory concentration (IC_50_) of RF in inhibiting expression of these inflammatory markers was 1462.18, 429.54, 570.16, 164.44, 402.72, 2333.46, 121.34, 3749.73, 261.22, 264.24, 364.75, 1733.80, 2167.70, 2167.70, 433.51, and 138.68 µg/mL for NO, intracellular calcium, IL-6, MCP-1, TNF-α, G-CSF, GM-CSF, IP-10, LIF, LIX, M-CSF, MIP-1α, MIP-1β, MIP-2, RANTES, and VEGF, respectively.

Our data indicate that RF may be useful to relieve NO-aggravated vasodilation and cytokine storm in SIRS due to gram-negative bacterial infection. Additionally, RF significantly decreased the release of intracellular calcium, mRNA expression of *Chop*, and phosphorylation of p38 MAPK in LPS-stimulated RAW 264.7 cells, which led to the hypothesis that RF-mediated regulation of inflammatory mediators in LPS-treated RAW 264.7 macrophages might be achieved through ER stress-related CHOP activation.

**Figure 5:**
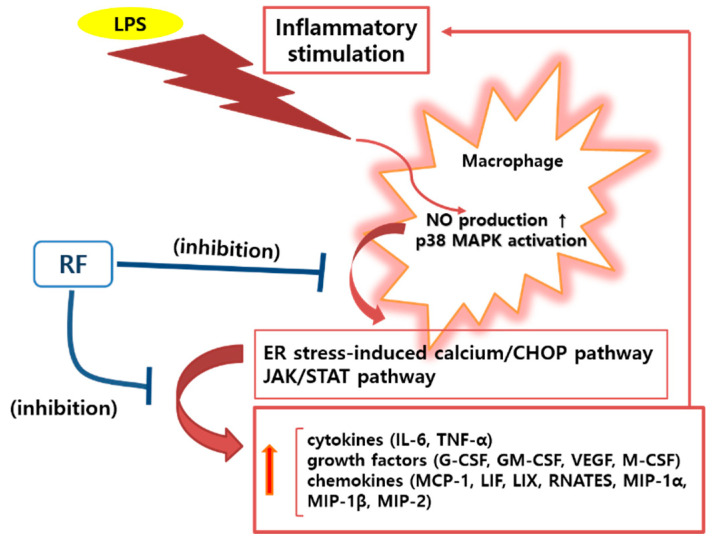
A schematic diagram of the immunoregulatory activity of Rubi Fructus water extract (RF) on lipopolysaccharide (LPS)-stimulated RAW 264.7 macrophages. RF alleviates LPS-stimulated macrophage activation via an ER stress-induced calcium/CHOP signaling pathway and the JAK/STAT pathway.

Many studies have reported ER stress-related calcium release and CHOP expression in stressed cells. In 1996, Wang and Ron reported that CHOP was known to be activated by p38 MAPK in stressed cells [38]. In 2006, Endo et al. reported that LPS stimulation of macrophages causes ER stress and CHOP expression, which initiated inflammasome activation and subsequent macrophage pyroptosis [39]. In 2007, Stout et al. reported that ER calcium stores were reduced and intracellular calcium concentration was initially increased during an inflammatory signaling cascade [40]. Mori also reported that NO depletes ER calcium, causes ER stress-induced CHOP expression, and leads to apoptosis [41]. In 2009, Timmins et al. reported that ER stress-induced calcium release from the ER lumen activates CAMK2a, which might enable macrophage apoptosis via Fas induction and/or activation of Stat1 in macrophages [42]. Tabas et al. also reported that ER-stress-induced CHOP activation, resulting in a release of ER calcium stores into the cytoplasm, and that cytoplasmic calcium activates CAMK2a, which in turn activates a number of pro-apoptotic processes [5]. In 2011, Cho et al. reported that COX-2 is an important mediator of the inflammatory response related to ER stress [43]. In 2017, Lee et al. reported that LPS induces expression of iNOS, COX-2, NO, TNF-α, and IL-6 via p38 MAPK and JAK/STAT signaling pathways in RAW 264.7 macrophages [44]. Guha and Mackman reported that p38 MAPK signaling in LPS-stimulated macrophages can activate a variety of transcription factors, including AP-1 (c-Fos/c-Jun) [45]. In the current study, RF was shown to reduce mRNA expression of *Camk2a*, *Stat1*, *Stat3*, *Jak2*, *Fas*, *c-Jun*, *c-Fos*, *Nos2*, and *Ptgs2* in LPS-stimulated RAW 264.7 macrophages. These results suggest that RF alleviates LPS-stimulated macrophage activation via ER stress-induced calcium/CHOP pathway and the JAK/STAT pathway, resulting in reduced production of inflammatory mediators such as NO, cytokines, chemokines, and growth factors (Figure 5).

However, this study could not determine the exact source from which intracellular calcium is released. It remains for future studies to elucidate whether the intracellular calcium level is increased through the influx of extracellular calcium or through depletion of ER calcium stores in LPS-stimulated RAW 264.7 macrophages. Moreover, we could not evaluate effects of RF on IL-1β production, phosphorylation of JNK, NF-κB activation, and ROS production in LPS-stimulated RAW 264.7. More detailed research will clarify the efficacy of RF in treating bacterial infectious diseases.

## Figures and Tables

**Table 1:** Primers used for quantitative PCR.

Name ^1^	Forward Primer (5′–3′)	Reverse Primer (5′–3′)
*Chop*	CCACCACACCTGAAAGCAG	TCCTCATACCAGGCTTCCA
*Camk2a*	AGCCATCCTCACCACTAT	ATTCCTTCACGCCATCATT
*Stat1*	TGAGATGTCCCGGATAGTGG	CGCCAGAGAGAAATTCGTGT
*Stat3*	GTCTGCAGAGT TCAAGCACCT	TCCTCAGTCACGATCAAGGAG
*Jak2*	TTGGTTTTGAATTATGGTGTCTGT	TCCAAATTTTACAAATTCTTGAACC
*Fas*	CGCTGTTTTCCCTTGCTG	CCTTGAGTATGAACTCTTAACTGTGAG
*c-Jun*	ACTGGGTTGCGACCTGAC	CAATAGGCCGCTGCTCTC
*c-Fos*	AGAGCGGGAATGGTGAAGA	TCTTCCTCTTCAGGAGATAGCTG
*Nos2*	TGGAGGTTCTGGATGAGAGC	AATGTCCAGGAAGTAGGTGAGG
*Ptgs2*	TCAAACAGTTTCTCTACAACAACTCC	ACATTTCTTCCCCCAGCAA
*β-actin*	CTAAGGCCAACCGTGAAAAG	ACCAGAGGCATACAGGGACA

^1^ Primer’s names; C/EBP homologous protein (*Chop*), calcium/calmodulin dependent protein kinase II alpha (*Camk2a*), signal transducers and activators of transcription 1 (*Stat1*), *Stat3*, Janus kinase 2 (*Jak2*), first apoptosis signal receptor (*Fas*), c-Jun, c-Fos, nitric oxide synthase 2 (*Nos2*), prostaglandin-endoperoxide synthase 2 (*Ptgs2*), and *β-actin*.

## Abbreviations

RF	Water extract of Rubi Fructus
LPS	Lipopolysaccharide
NO	Nitric Oxide
PGE2	Prostaglandin E2
IL	Interleukin
MCP	Monocyte chemotactic activating factor
TNF	Tumor necrosis factor
IgG2b	Immunoglobulin G2b
OD	Optical density
LIX	Lipopolysaccharide-induced CXC chemokine
LIF	Leukemia inhibitory factor
G-CSF	Granulocyte colony-stimulating factor
GM-CSF	Granulocyte macrophage colony-stimulating factor
VEGF	Vascular endothelial growth factor
M-CSF	Macrophage colony-stimulating factor
MIP	Macrophage inflammatory protein
RANTES	Regulated on activation, normal T cell expressed and secreted
IP	Interferon gamma-induced protein
ER	Endoplasmic reticulum
CHOP	C/EBP homologous protein
CAMK2a	Calcium/calmodulin dependent protein kinase II alpha
MAPK	Mitogen-activated protein kinase
JAK	Janus kinase
STAT	Signal transducers and activators of transcription
FAS	First apoptosis signal receptor
NOS	Nitric oxide synthase
PTGS	Prostaglandin-endoperoxide synthase
SIRS	Systemic inflammatory response syndrome
RE	Rutin equivalents
ERF	Ethanol extract of Rubi Fructus
WRF	Water extract of Rubi Fructus
DMEM	Dulbecco’s modified Eagle’s medium
HMSB	Handheld Magnetic Separation Block
SB	Staining Buffer
Nor	Normal group
MODS	Multiple organ dysfunction syndrome
JNK	Jun NH2-terminal kinase
NF	Nuclear factor
iNOS	Inducible nitric oxide synthase
COX-2	Cyclooxygenase 2
IC_50_	Half maximal inhibitory concentration
ROS	Reactive oxygen species
